# Molecular profiling and bioactive potential of an endophytic fungus *Aspergillus sulphureus* isolated from *Sida acuta*: a medicinal plant

**DOI:** 10.1080/13880209.2017.1315435

**Published:** 2017-04-20

**Authors:** M. Murali, C. Mahendra, P. Hema, N. Rajashekar, A. Nataraju, M. S. Sudarshana, K. N. Amruthesh

**Affiliations:** a Applied Plant Pathology Laboratory, Department of Studies in Botany, University of Mysore, Mysuru, India;; b Department of Botany, F.M.K.M. Cariappa College, Madikeri, India;; c Department of Biochemistry, Karnataka State Open University, Mysuru, India

**Keywords:** Antibacterial, antioxidant, cytotoxicity, endophyte, haemolytic

## Abstract

**Context:** *Sida* acuta Burm.f. (Malvaceae) extracts are reported to have applications against malaria, diuretic, antipyretic, nervous and urinary diseases. No fungal endophytes of *S. acuta* are reported.

**Objective:** Isolation, identification and evaluation of antibacterial, antioxidant, anticancer and haemolytic potential of fungal endophytes from the ethnomedcinal plant *S. acuta*.

**Materials and methods:** *Sida acuta* stem segments were placed on PDA medium to isolate endophytic fungi. The fungus was identified by genomic DNA analysis and phylogenetic tree was constructed using ITS sequences (GenBank) to confirm species. The antibacterial efficacy of *Aspergillus sulphureus* MME12 ethyl acetate extract was tested against Gram-positive and Gram-negative pathogenic bacteria. DPPH free radical scavenging activity, anticancer and DNA fragmentation against EAC cells, and direct haemolytic activity (100–500 μg/mL) using human erythrocytes were determined.

**Results and discussion:** The ethyl acetate extract of *A. sulphureus* (Fresen.) Wehmer (Trichocomaceae) demonstrated significant antibacterial potential against *Staphylococcus aureus, Bacillus subtilis, Escherichia coli* and *Salmonella typhi* compared to streptomycin. MIC against test pathogens was in the range of 15.6–62.5 μg/mL. The antioxidant results revealed significant RSA from 12.43% to 62.02% (IC_50_ = 350.4 μg/mL, *p* ≤ 0.05). MME12 offered considerable inhibition of EAC proliferation (23% to 84%, IC_50_ = 216.7 μg/mL, *p ≤* 0.05) supported by DNA fragmentation studies. The extract also offered insignificant haemolysis (5.6%) compared to Triton X-100.

**Conclusions:** A single endophytic fungus, *A. sulphureus* MME12 was isolated and identified using molecular profiling. The above-mentioned findings support the pharmacological application of *A. sulphureus* MME12 extract and demand for purification of the active principle(s).

## Introduction

Natural products are a continuous source of new lead compounds, which can be utilized in various fields including pharmacy and agriculture. Unlike environmentally hazardous synthetic compounds, bioactive natural products retain an immense impact on modern medicine and agriculture (Deepika et al. 2016). Isolation of endophytes have carried out in a variety of plant species including those growing under extreme conditions, as the global diversity of endophytic fungi are limitless (Khan et al. 2011). However, still a large number of ecosystems need to be explored for such diversified endophytes and their medicinally important bioactive metabolites (Strobel 2006; Dutta et al. 2014; Zheng et al. 2016).

A comprehensive study has indicated that 51% of bioactive substances isolated from endophytic fungi were previously unknown (Schulz & Boyle 2005). Therefore, currently endophytic fungi are extensively explored for their ability to produce antimicrobial, anticancer, antioxidants, immunosuppressive compounds, in addition protect their host against various phytopathogens (Schulz & Boyle 2005; Saikkonen et al. 2006; Khan et al. 2011; Kumar et al. 2013; Dutta et al. 2014; Murali et al. 2016; Zheng et al. 2016). Endophytic fungi are also known to mimic the chemistry of their host plants and produce the same bioactive natural products, suggesting the possibility of inter-generic genetic exchange between the plant and the fungus (Kumar et al. 2013; Deepika et al. 2016).

*Sida acuta* Burm.f. (Malvaceae) is a medicinal plant with a broad range of phytochemicals such as tannins, saponins, flavonoids, alkaloids, organic acids and anthraquinones (Akaneme 2008), while the stem constitutes only tannins, alkaloids and anthraquinones (Edeoga et al. 2005). *Sida acuta* extracts find foremost applications against malaria, diuretic, antipyretic, nervous and also against urinary disease (Karou et al. 2007). Interestingly, no fungal endophytes of *S. acuta* are reported. Further, the metabolic constituents and their pharmacological applications need to be systematically explored. Therefore, the current study was conducted to isolate endophytic fungi from *S. acuta* and to evaluate their potential antibacterial, antioxidant and anticancer activities.

## Materials and methods

### Collection of plant material

The healthy and mature stem part of *S. acuta* was collected from Mysore region [Rainy season (July–September 2013)] (12.3°N 76.6°E, elevation 776 m), Karnataka, India and brought to the laboratory for immediate processing. The collected plant was identified by a Taxonomist (Prof. M.S. Sudarshana), Department of Studies in Botany, University of Mysore, Mysuru and authenticated with the help of Flora of Presidency of Madras (Gamble 1935). A Voucher Specimen No. MM2016 is deposited in the Herbarium, Department of Studies in Botany, University of Mysore, Manasagangotri, Mysore, Karnataka, India.

### Isolation and identification of endophytic fungi

The collected stem samples were cut into small pieces (1–2 cm length) under aseptic conditions using a sterile scalpel and were subjected to surface sterilization by sequential steps (Rakshith et al. 2013). After sterilization, 8–10 stem segments were placed on Petri plates containing 20 mL of potato dextrose agar (PDA) medium supplemented with antibiotic chloramphenicol to avoid the emergence of endophytic bacteria. The Petri plates were sealed using Parafilm and incubated at 25 ± 2 °C for 3 weeks. Endophytic fungal colonies emerging from their host were sub-cultured onto the Petri plates containing PDA devoid of antibiotic to obtain pure cultures. The fungi were identified based on their morphological and cultural characteristics.

### Molecular profiling of Aspergillus sp

#### Genomic DNA extraction

Genomic DNA was extracted from the lyophilized fungal mat of *Aspergillus* sp. MME12 by the cetyltrimethylammonium bromide (CTAB) method. The nuclear ribosomal DNA and internally transcribed spacer (ITS) region were amplified using ITS1 (5′- TCCGTAGGTGAACCTGCG-3′) and ITS4 (5′-TCCTCCGC-TTATTGATATG-3′) primers (White et al. 1990). The PCR conditions include; initial denaturation (95 °C for 10 min); 35 cycles [95 °C for 1 min, 55 °C for 1 min, 72 °C for 2 min] and a final extension at 72 °C for 8 min. The amplicon was sequenced using ITS1 and ITS4 primers in two different sequencing reactions. A contiguous sequence out of two sequences was generated using CAP3 sequence assembly program (Huang & Madan 1999) and submitted to the GenBank of the National Center for Biotechnology Information (NCBI).

#### Phylogenetic analysis

The phylogenetic tree was constructed using ITS sequences retrieved from GenBank included marine isolates of *A. sulphureus*, MME12 and other closely related *Aspergillus* spp. A distantly related isolate *A. tubingensis* was also used as an outgroup during analysis. The multiple sequence alignment was generated using the Clustal W version 1.7 (MSA) program. The phylogenetic dendrogram was generated by MEGA 6.0 software with a bootstrap consensus of 1000 replicates (Padhi & Tayung 2013).

#### Fermentation of endophytic fungus

Five to ten discs (10 mm) of *A. sulphureus* MME12 on PDA were picked and inoculated in 1000 mL Erlenmeyer flasks containing 500 mL of potato dextrose broth (PDB) for 4-6 weeks at 25 ± 2 °C under static conditions devoid of antibiotic. After incubation, the culture broth was filtered using Whatman No. 1 filter paper and the resulting culture filtrate was centrifuged at 5000 rpm for 10 min. The obtained supernatant was extracted with ethyl acetate and evaporated to dryness using flash evaporator. As ethyl acetate is nearly a polar solvent and immiscible with water during liquid–liquid extraction of secondary metabolites using separating funnel, it has been extensively used in endophyte extraction studies (Strobel et al. 1996; Schulz & Boyle 2005; Buatong et al. 2011).

#### Antibacterial activity

The antibacterial screening of the ethyl acetate extract was carried out by agar plug and disc diffusion method (Clinical and Laboratory Standards Institute (CLSI, 2012b)) against Gram-positive (*Staphylococcus aureus* MTCC 7443 and *Bacillus subtilis* MTCC 121) and Gram-negative (*Escherichia coli* MTCC 7410 and *Salmonella typhi* MTCC 733) bacteria obtained from Microbial Type Culture Collection and Gene Bank (MTCC), Institute of Microbial Technology, Chandigarh, India. About 100 μL of the test bacteria (1.5 × 10^8^ CFU mL^−1^) were seeded uniformly onto the surface of NA media using a sterile glass spreader. The sterile discs (6 mm) were loaded with 50 μL of fungal extract (100 μg disc^−1^) and placed on NA plates. The inoculated plates were incubated at 37 ± 2 °C for 24 h and zone of inhibition around the discs were measured. The experiment was carried out in triplicates with appropriate controls.

#### Thin-layer chromatography and bioautography

The extract of *A. sulphureus* MME12 was subjected to TLC and agar overlay bioautography (Valgas et al. 2007). About 10 μL of ethyl acetate extract was spotted on pre-coated TLC silica gel plates (TLC Silica gel 60 F_254_, Merk, Germany) and air dried. The spotted TLC plates were eluted in an elution system of hexane and ethyl acetate (1:1). The developed chromatogram was air dried and observed in a UV chamber at 254 nm and 365 nm for clearly resolved bands and the corresponding *R_f_* (Retention factor) values were calculated. The chromatograms were subjected to agar overlay bioautography against *E. coli* and the plates were incubated at 37 ± 2 °C for 24 h and observed for inhibition zone. The experiment was repeated thrice.

#### Minimum inhibitory concentration (MIC)

Minimal Inhibitory Concentration (MIC) was determined by broth microdilution technique (CLSI 2012a). The ethyl acetate extract was diluted to a concentration of 40 mg/mL, which served as a stock solution. Broth (100 μL) was dispensed into each well and a twofold serial dilution was carried out (2–0.0009 mg/mL). A 10 μL inoculum suspension was added to each well and incubated at 37 ± 2 °C for 24 h. Absorbance was measured at 620 nm using ELISA plate reader (LabTech 4000). MIC was also detected by adding 10 μL/well of TTC (2,3,5-triphenyl tetrazolium chloride at 2 mg/mL) and incubated for 30 min. The lowest concentration at which the colour change occurred was taken as the MIC value. All MIC tests were carried out in triplicates.

#### Antioxidant activity

The DPPH free radical scavenging activity (RSA) of *A. sulphureus* MME12 extract was determined by DPPH method (Sultanova et al. 2001). Different concentrations (100–500 μg/mL) of test sample were prepared, while the concentration of DPPH remained same. The reaction mixture containing 5 μL of extract and 95 μL of DPPH (300 μM) in methanol was incubated at 37 ± 2 °C for 30 min and the absorbance was measured at 517 nm. The experiment was repeated three times with ascorbic acid (HiMedia, Mumbai, India) as standard. The percent RSA was calculated by comparing with appropriate positive and negative controls.

$$\%\;RSA=\frac{Absorbance\;of\;control-Absorbance\;of\;sample}{Absorbance\;of\;control}\times 100$$

#### Anticancer and DNA fragmentation assay

Short-term cytotoxicity was assessed by Trypan blue exclusion method (Gupta 2002). The *in vitro* cytotoxicity potentials of *A. sulphureus* MME12 extract was determined at various concentrations (100, 200, 300, 400 and 500 μg/mL) by dissolving it in 10% dimethyl sulfoxide. All the concentrations of the extract were mixed with 1 mL of Ehrlich Ascitic Carcinoma (EAC) cells (1 × 10^4^ cells/mL) and incubated at 37 ± 2 °C for 4 h. After incubation, the Trypan blue dye (10 μL) was added to all the vials and the number of dead cells was counted using a haemocytometer. The cells that are viable exclude Trypan blue, while the uptake of trypan blue can be noticed in dead cells. The experiment was repeated three times with curcumin (Sigma-Aldrich, Bengaluru, India) as standard and the percentage of cytotoxicity was calculated.

$$\%\;Cell\;death=\frac{No.\;of\;dead\;cells}{No.\;viable\;cells+No.\;of\;dead\;cells}\times 100$$

The DNA fragmentation assay was carried out according to the method by McGahon et al. (1995) with slight modifications. EAC cells (1 × 10^4^ cells/mL) were incubated at 37 ± 2 °C for 4 h with *A. sulphureus* MME12 extract at a concentration of 500 μg/mL. Following incubation, the cell suspension was centrifuged at 2000 *g* (5 min at 4 °C) and DNA was isolated from pellets using DNA purification kit (HiMedia). Extracted DNA was dissolved in 50 μL TE buffer and electrophoresed on 1% agarose gel containing ethidium bromide and visualized under Gel-Documentation. The experiment was repeated three times.

#### Haemolytic assay

Haemolytic activity was carried out according to the method of Pagliara and Caroppo (2012). In brief, about 10 mL of blood was collected from healthy volunteers following consent and was centrifuged at 3000 rpm for 3 min and the supernatant was discarded. Erythrocytes were washed three times using Dulbecco’s phosphate buffer saline (D-PBS) solution following centrifugation. The final pellet was diluted to 1:24 (v/v) in D-PBS, pH 7.0 containing 0.5 mM boric acid and 1 mM calcium chloride and brought to a final concentration of 5% (v/v).

For the haemolytic assay, various concentrations of endophytic extract (in triplicates) were incubated with erythrocytes suspension at 37 °C for 1 h. The free haemoglobin content in the supernatant was measured spectrophotometrically at 540 nm. D-PBS and 1% Triton X-100 (HiMedia, India) served as negative and positive controls, respectively. The experiment was repeated thrice and the per cent haemolytic activity was calculated using the formula:

$$\%\;Haemolysis=\frac{Absorbance\;of\;test\;sample-Absorbance\;of\;DPBS\;}{Absorbance\;of\;DPBS-Absorbance\;of\;1\%\;Triton\;X-100}\times 100$$

#### Statistical analysis

Data from three replicates were analyzed for each experiment and analysis of variance (ANOVA) using SPSS Inc. 16.0. Significant effects of treatments were determined by *F* values (*p* ≤ 0.05). Tukey’s HSD test separated treatment means.

## Results and discussion

### Isolation and identification of endophytic fungi

**Figure 1.**
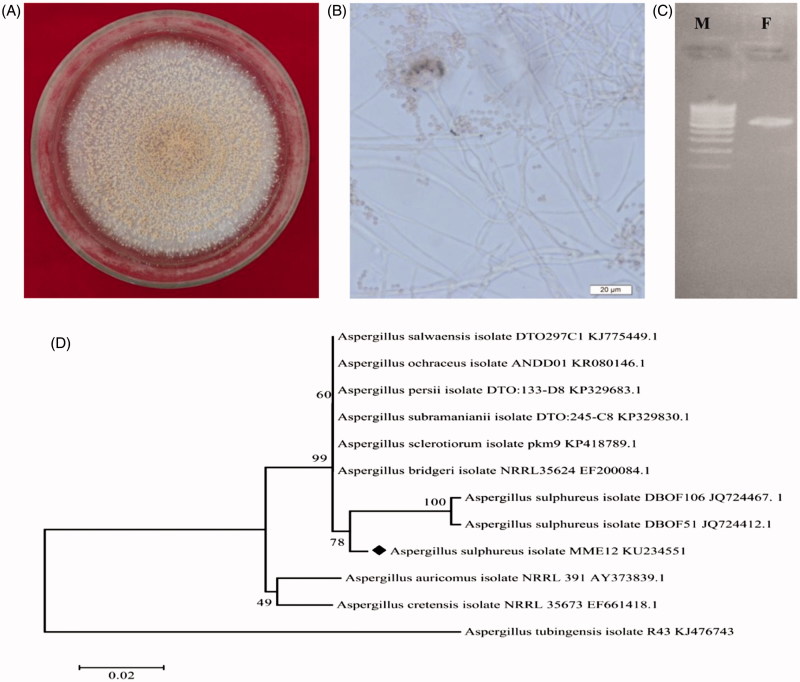
Characterization of endophytic fungus *A. sulphureus* MME12. **(**A) Colony and (B) conidial morphology of *Aspergillus* sp. MME12; (C) electropherograms of PCR amplification of *A. sulphureus* MME12; (D) Phylogenetic tree derived from Neighbour Joining analysis showing the evolutionary relationship of *A. sulphureus* MME12 with its closest BLAST hits. M: DNA marker; F: *A. sulphureus* MME12.

A single endophytic fungus was isolated from *S. acuta* stem tissue, and no other fungal colonies emerged following imprint PDA plates that demonstrate active surface sterilization and true endophyte isolation. Similar, standardized endophytic fungal isolation by imprint method has been used in *Ficus pumila*, *Basella rubra* and *Camellia sinensis* to evaluate the effectiveness of surface sterilization of the plant materials (Rakshith et al. 2013; Hema et al. 2015; Guo et al. 2016). The results of the present study are in agreement with the findings of Rao et al. (2015), where a single endophytic fungus *Gliomastix polychrome* was isolated from surface-sterilized bark of *Combretum latifolium*, while there are also reports of isolation of more than one endophytic fungi from *Nymphaea nouchali* (Dissanayake et al. 2016). Our fungal isolate emerging from the tissue of the inoculated plant part was identified as *Aspergillus* sp. MME12 by prominent morphological and cultural characteristics such as powdery texture, sulphur yellow with fewer spores on the surface and brownish on the reverse side with reduced sporulation (Figure 1(A,B)).

### Molecular profiling of *Aspergillus* sp. MME12

#### Genomic DNA extraction and phylogenetic analysis

The PCR amplification of the isolated DNA from *Aspergillus* sp. MME12 with specific primers generated a band ranging from 550–600 base pair for the ITS region (Figure 1(C)). The ITS rDNA profiling is a standard and cost-effective method to identify the complex microbial communities in species level at exceptional depth and resolution following morphological and cultural examination (Rastogi & Sani 2011; Rao et al. 2015). Therefore, based on the ITS sequences, we identified the *Aspergillus* sp. MME12 as *A. sulphureus*. The DNA fragments were sequenced separately and deposited in NCBI-GenBank under the accession number KU234551. The ITS sequence of *A. sulphureus* MME12 (∼559 bp) revealed high similarity (99% sequence identity, *E*-value = 0) with the marine fungus *A. sulphureus* (JQ724467 and JQ724412). Further, phylogenetic analysis using NJ tree with 1000 bootstraps showed clustering based on species and ecological characters (Figure 1(D)). The alignment of the obtained ITS sequence with those of NCBI database resulted in identifying closely related sequences and NJ tree firmly demonstrated that *A. sulphureus* MME12 fell into the cluster of a marine *A. sulphureus*.

#### Antibacterial activity

**Figure 2.**
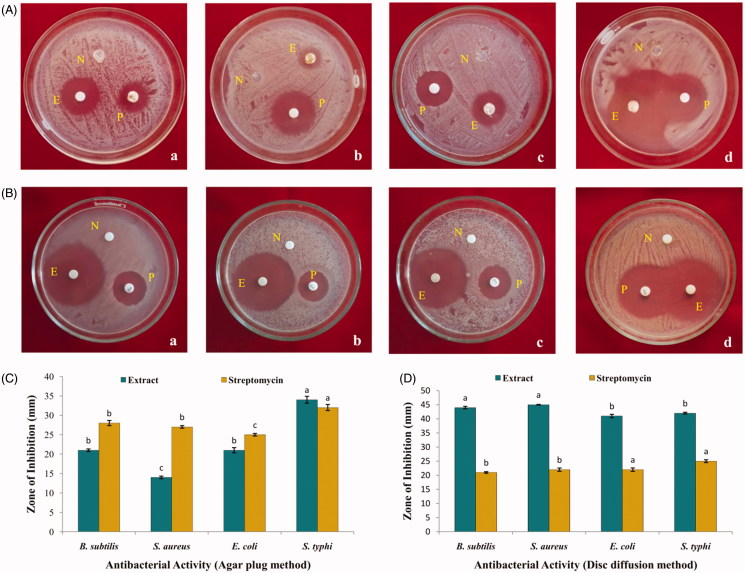
Antibacterial activity of ethyl acetate extract of *A. sulphureus* MME12. (A) and (C) Agar plug method; (B) and (D) Disc diffusion method. N: Negative control; P: Positive control; E: *A. sulphureus* extract; a: *B. subtilis*; b: *Staph. aureus*; c: *E. coli*; d: *S. typhi*.

The ethyl acetate extract of *A. sulphureus* MME12 was evaluated for antibacterial potential against a wide range of multi-drug-resistant Gram-positive (*S. aureus* and *B. subtilis*) and Gram-negative (*E. coli* and *S. typhi)* human pathogens by agar plug and disc diffusion methods. In agar plug method, the antibacterial activity zone of the *A. sulphureus* MME12 was comparable to standard control streptomycin (Figure 2(A, C)). Similarly, the results of antibacterial activity by disc diffusion assay offered 41–45 mm zone of inhibition against test pathogens (Figure 2(B,D)). Ramasamy et al. (2010), with the ethyl acetate extracts of two endophytic *Aspergillus* sp. have shown parallel results against *B. subtilis*, *E. coli*, *Micrococcus luteus*, *Pseudomonas aeruginosa* and *S. aureus*. Likewise, Rao et al. (2015) and Dissanayake et al. (2016) reported significant antibacterial potential of endophytic fungus isolated from *Combretum latifolium* and *Nymphaea nouchali*, respectively.

#### TLC-bioautography

**Figure 3.**
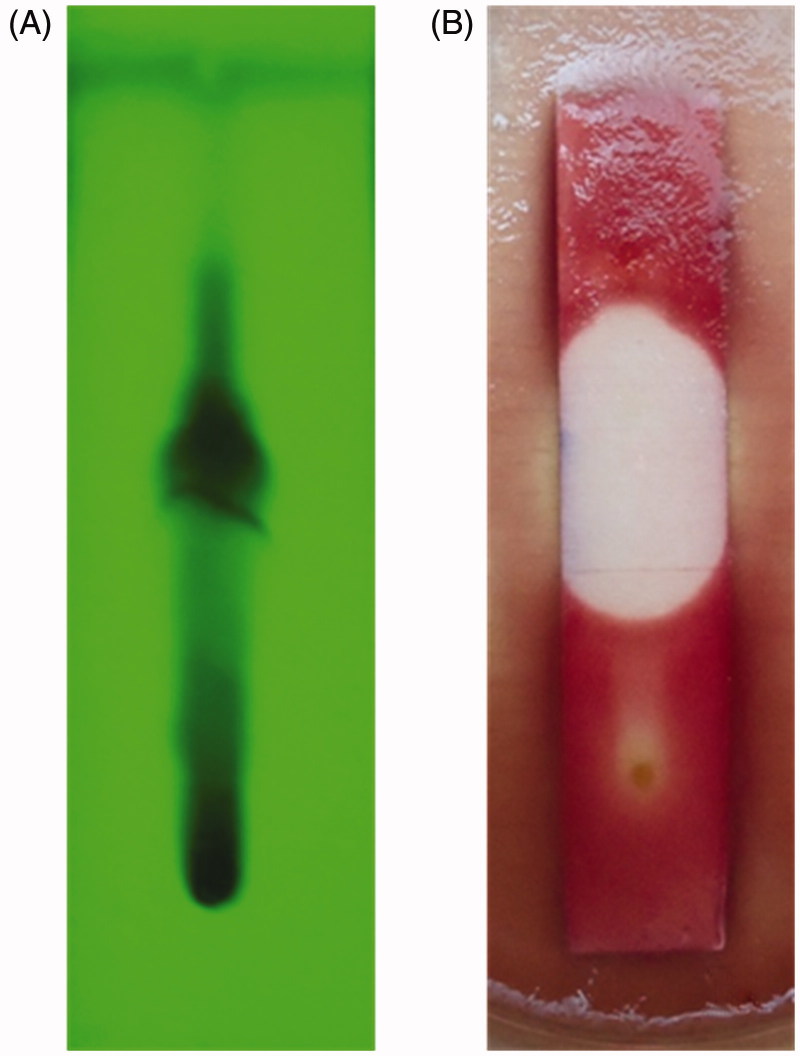
TLC-bioautography of ethyl acetate extract of *A. sulphureus* MME12. (A) TLC chromatogram under short UV- light; (B) zone of inhibition against *E. coli*.

TLC-bioautography is considered to be one of the simplest and reproducible methods for the isolation of active metabolites from natural products (Patra et al. 2012; Rao et al. 2015; Balouiri et al. 2016). A TLC-based bioautography was carried out to separate the active metabolites of ethyl acetate extract of *A. sulphureus* MME12 and examine their antibacterial potential following separation. Hexane and ethyl acetate solvent system in the ratio of 1:1 was optimized based on pilot experiments. A total of 10 bands with *R*_f_ values of 0.07, 0.15, 0.25, 0.33, 0.47, 0.52, 0.57, 0.71, 0.77 and 0.88 were obtained (Figure 3(A)). A dark spot at the *R*_f_ value of 0.52 in TLC showed significant antibacterial potential with an effective inhibition zone against the test pathogen (Figure 3(B)). Correspondingly, TLC-bioautography has been used to check the antimicrobial potential of separated fractions of ethyl acetate extract of endophytic fungi (Rakshith et al. 2013; Rao et al. 2015).

#### Minimum inhibitory concentration (MIC)

**Figure 4.**
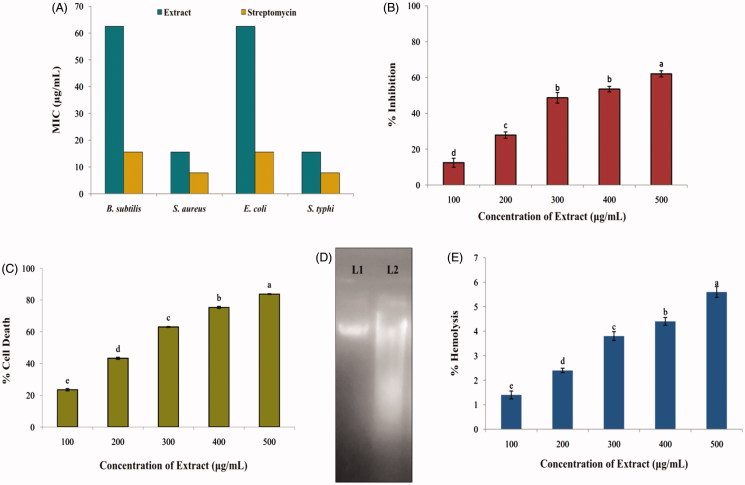
Bioactive potential of ethyl acetate extract of *A. sulphureus* MME12. (A) MIC against test pathogens; (B) DPPH radical scavenging activity; (C) cytotoxicity against Ehrlich Ascitic Carcinoma cells; (D) haemolytic activity against human erythrocytes; (E) DNA damage study against EAC cells. L1: DNA of EAC cells; L2: DNA of EAC cells treated with extract of *A. sulphureus* MME12. Each value is the mean for three replicates (*n* = 3) and bars sharing the same letters are not significantly different (*p* ≤ 0.05) according to Tukey’s HSD (honest significant difference). The vertical bar indicates the standard error.

MIC results of *Aspergillus* sp. MME12 ethyl acetate extract varied in the range of 15.6–62.5 μg/mL. The results revealed MIC of 15.6, 62.5, 15.6 and 62.5 μg/mL against *S. aureus*, *B. subtilis*, *S. typhi* and *E. coli*, respectively (Figure 4(A)). Likewise, ethyl acetate extract of endophytic fungi from *Bacopa monnieri* displayed antimicrobial activity against *B. subtilis*, *P. aeruginosa*, *S. typhimurium*, *E. coli*, *K. pneumonia* and *S. aureus* with MIC of 10–100 μg/mL (Katoch et al. 2014). Also, Ratnaweera et al. (2015) and Dissanayake et al. (2016) have reported that endophytic fungi *Fusarium* sp. and *Chaetomium globosum* isolated from *Opuntia dillenii* and *N. nouchali*, respectively, offered antibacterial activity with MIC ranging from 8 to 32 μg/mL against *B. subtilis*, *S. aureus* and methicillin-resistant *S. aureus*.

#### Antioxidant activity by DPPH method

The ethyl acetate extract of MME12 was examined for its antioxidant activity by DPPH free Radical Scavenging Activity (RSA) method. The results revealed significant (*p* ≤ 0.05) RSA in *A. sulphureus* MME12 extracts from 12.43% to 62.02% (Figure 4(B)), with an IC_50_ at 350.4 μg/mL, while ascorbic acid offered 77% inhibition at 50 μg/mL. Khiralla et al. (2015) isolated endophytic *Aspergillus* sp. from *Trigonella foenum-graecum* seeds, which provided antioxidant activity with an IC_50_ value of 18 μg/mL. Further, potent antioxidant activity has been observed in endophytic fungi isolated from *Mimosa pudica, Tridax procumbens* and *Eugenia jambolana* (Sharma & Sharma 2010; Yadav et al. 2014), which supports our findings.

#### Anticancer and DNA fragmentation assay

Cytotoxicity of *A. sulphureus* MME12 extract was determined against EAC cells using Trypan blue exclusion method by counting viable and non-viable cells in a haemocytometer. *A spergillus sulphureus* MME12 extract offered significant (*p ≤* 0.05) inhibition of the proliferation of EAC cells with inhibition per cent ranging from 23% to 84% among the tested concentrations (Figure 4(C)), while the standard curcumin offered 87% inhibition at 10 μg/mL. The test extract was found to have an IC_50_ value of 216.7 μg/mL. Correspondingly, an endophytic fungus similar to *Phoma* sp. isolated from *Cinnamomum mollissimum* offered potent cytotoxicity against murine leucemia cells (Santiago et al. 2012). Also, the ethyl acetate extract of endophytic fungi isolated from *P. crocatum* and red alga *Bostrychia tenella* inhibited the growth of different tumour cell lines with IC_50_ value of 37.43 (T47D) and 1 μg/mL (SF-295), respectively (Astuti et al. 2014; de Felicio et al. 2015). It was also noted that EAC cells without the treatment of extract had no cytotoxicity effect in the present study. Further, the result of the DNA fragmentation assay evidences the influence of cytotoxicity due to damage of DNA in EAC cells treated with the extract (Figure 4(D)). The results are in corroboration with earlier findings of Ruma et al. (2013), as DNA damage in super-coiled pBR322 plasmid cells were noticed upon treatment with the extracts of endophytic fungi. The above results clearly suggest that *A. sulphureus* MME12 extract is cytotoxic to cancer cells through DNA damage.

#### Haemolytic assay

Our findings have demonstrated the antioxidant and antimicrobial potential of *A. sulphureus* MME12 extract against most common and lethal human bacteria. Also, the anticancer and DNA damage study indicates its anticancer applications. Considering its potential, it becomes significantly relevant to determine the cytotoxicity of the extract against normal cells. Therefore, we determined the direct haemolytic activity using normal human erythrocytes. The extract was tested at various doses from 100–500 μg/mL in comparison to 1% Triton X-100. Encouragingly, the extract even at its maximum dose tested demonstrated insignificant haemolysis. The haemolysis was found to be 1.4 ± 0.8 to 5.6 ± 1.2% at 100–500 μg/mL concentrations, respectively, compared to 100% haemolysis in 1% Triton X-100, a known haemolytic agent (Figure 4(E)). Recent studies by Li et al. (2016) showed the anticancer activity of an extract from *B. ochroleuca* M21 against liver (HepG2), gastric (SGC-7901) and colon (HT29) cancer cell lines in concentration ranges of 0.1–0.45 mg/mL. However, the extracts did not have cytotoxic activity on normal human liver HL-7702 cells at concentrations of 0.025–1.6 mg/mL indicating their selectivity for cancer cells. Along similar lines, our data further confirms the biological application of *A. sulphureus* MME12 extract for applications in pharmaceutical fields without harming the healthy cells of the body.

## Conclusions

The present work is the first report on the incidence of endophytic fungi *A. sulphureus* inhabiting *S. acuta*. The ethyl acetate extract from *A. sulphureus* MME12 demonstrated significant antibacterial potential, antioxidant and anticancer activity comparable to the standard drugs. Further, the toxicity studies of the extract tested against human erythrocytes using a haemolytic assay show their nontoxic nature. The above results demand for purification of the novel active principle(s) from *A. sulphureus* MME12 and determination of their pharmacological application.

## Supplementary Material

Amruthesh_KN_et_al_supplemental_content.zip
