# Supplementary material for: Molecular profiling and bioactive potential of an endophytic fungus Aspergillus sulphureus isolated from Sida acuta: a medicinal plant
Source: Pharm Biol. 2017 Apr 20;55(1):1623–30. doi: 10.1080/13880209.2017.1315435 (PMC7012020; doi:10.1080/13880209.2017.1315435)
Supplement: Amruthesh_KN_et_al_supplemental_content.zip [file IPHB_A_1315435_SM6143.zip › Amruthesh KN et al supplemental content.docx]

**
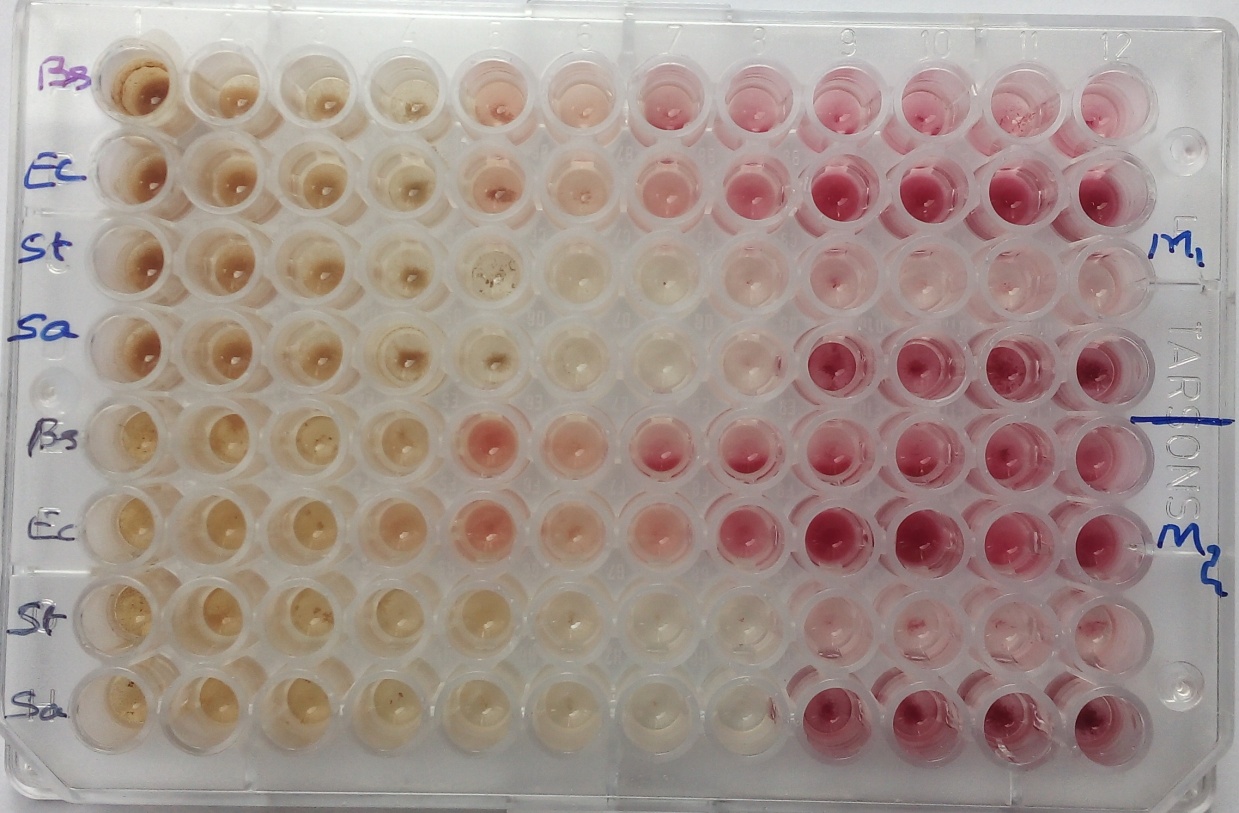
**

**Supplementary Figure 1:** Minimum Inhibitory Concentration (MIC) of *A. sulphureus* MME12 ethyl acetate extract against test pathogens. Bs- *B. subtilis*; Ec- *E. coli*; St- S*. typhi*; Sa- *Staph. aureus*; M_1_- Replicate 1; M_2_- Replicate 2

**Supplementary Figure 2:** Cytotoxicity of ethyl acetate extract of *A. sulphureus* MME12 against EAC cells. A- Control; B- Treated with *A. sulphureus* MME12 (500 µg/mL)
